# Smoking disrupts the relationship between cerebrospinal fluid IL-1β and multiple subdimensions of sleep

**DOI:** 10.1016/j.bbih.2025.100987

**Published:** 2025-03-26

**Authors:** Yueling Hu, Siyuan Li, Yingjie Chen, Xingguang Luo, Yu-Hsin Chen, Yimin Kang, Weiming Hu, Li Chen, Siling Ye, Xinchen Zhou, Yanlong Liu, Fan Wang, Yuying Li

**Affiliations:** a School of Mental Health, Wenzhou Medical University, Wenzhou 325035, China; b Cixi Biomedical Research Institute, Wenzhou Medical University, Zhejiang, China; c Department of Psychiatry, Yale University School of Medicine, New Haven, CT 06510, USA; d Psychosomatic Medicine Research Division, Inner Mongolia Medical University, Hohhot, China; e Department of Psychiatry, The Third Hospital of Quzhou, Quzhou, China; f Beijing Huilongguan Hospital, Peking University, Beijing, China; g Ruian People's Hospital, Wenzhou Medical University Affiliated Third Hospital, Wenzhou, China

**Keywords:** IL-1β, Sleep, Cigarette smoking, Cerebrospinal fluid, Neuroinflammation

## Abstract

**Objective:**

Inflammatory factors and cigarette smoking have been associated with sleep disorders but molecular mechanisms that regulate sleep, specifically the role of interleukin-1β (IL-1β), remain controversial. Individuals’ cerebrospinal fluid (CSF) IL-1β, smoking behavior, and sleep data were collected to investigate how smoking may influence the relationship between CSF IL-1β and sleep via moderation analysis.

**Methods:**

A total of 191 Chinese male patients, including 104 non-smokers and 87 active smokers, scheduled for anterior cruciate ligament reconstructive surgery, were recruited. Pittsburgh Sleep Quality Index (PSQI) scores were collected prior to surgery, and CSF samples were obtained during preoperative lumbar puncture.

**Results:**

Active smokers compared to non-smokers exhibited higher scores across various subdimensions of PSQI, specifically poorer sleep quality, increased sleep latency, reduced sleep efficiency, and heightened sleep disturbance (all p < 0.05). Positive correlations were observed between CSF IL-1β levels and PSQI total scores, as well as several subdimensions of sleep (all p < 0.05) in non-smokers. In contrast, a negative correlation was observed between CSF IL-1β levels and sleep efficiency scores (p < 0.05) among active smokers. Moderation analysis revealed that smoking negatively moderated the relationship between CSF IL-1β and sleep, particularly in PSQI total scores (β = −0.95, p < 0.001), sleep latency scores (β = −1.05, p < 0.001), and sleep disturbance scores (β = −0.45, p < 0.05).

**Conclusions:**

The current study found that smoking disrupts multiple subdimensions of sleep and moderates the effect of the neuroinflammatory factor CSF IL-1β on PSQI sleep latency and sleep disturbance scores.

## 1 Introduction

Sleep disorders disrupt the natural circadian rhythm, adversely affecting both mental and physical well-being (Pigeon, 2010). Individuals with short sleep durations are more prone to mood and anxiety disorders (Grandner, 2017; Léger and Bayon, 2010; Zhao et al., 2020), as well as an elevated risk of cardiovascular events (Ridker et al., 2003) and hypertension (Gangwisch, 2014). A significant portion of the global population, estimated to be between one-third and one-fourth in industrialized nations and nearly half in China, has been reported varying degrees of sleep disorders (Ohayon, 2002). Recent findings report that sleep disorders are associated not only with alterations in inflammatory factors but also with lifestyle habits, such as smoking. Studies indicate a high prevalence of sleep-related complaints among cigarette smokers (Tian and Li, 2017; Zhang et al., 2006). Despite recent advancements in the molecular mechanisms that regulate sleep, the role of Interleukin-1β (IL-1β) amongst smokers and non-smokers warrants further investigation.

IL-1β is a potent pro-inflammatory cytokine that plays a crucial role in host-defense responses and is produced/secreted by various cell types (Lopez-Castejon and Brough, 2011; Tian and Li, 2017). Notably, IL-1β is involved in sleep regulation, enhancing activity in sleep-active neurons while inhibiting wake-active neurons (Liu et al., 2020). However, research on the effect of IL-1β on sleep has yielded conflicting results (Chen et al., 2018). Some studies suggest a protective role for IL-1β in sleep, while others indicate a positive correlation between sleep insufficiency and IL-1β levels (Wang et al., 2022b; Zhai et al., 2021). These associations notably differ between smokers and non-smokers, as demonstrated in a previous study that have reported a positive correlation between CSF IL-1β and sleep in non-smokers, but not in smokers (Liu et al., 2020). This disparity suggests that smoking may modulate the relationship between CSF IL-1β and sleep.

Previous research has reported that cigarette smokers are more prone to various sleep disturbances, including sleep-disordered breathing, insomnia, and poor sleep characterized by shorter durations and increased latency (Purani et al., 2019). Smoking is also associated with disrupted sleep patterns, characterized by difficulties falling asleep and frequent awakenings (Dall'Olio et al., 1994; Purani et al., 2019). Despite these findings, there remains a significant gap in research concerning the interplay between IL-1β, smoking, and their combined effects on sleep. While some studies focus on smoking-induced neuroinflammation, others report conflicting results regarding cytokine levels in smokers (Lee et al., 2012; Wang et al., 2022a).

Given these inconsistencies, further investigation into the interaction between smoking and IL-1β is warranted to advance research that could improve sleep health. Therefore, this study aims to explore the associations between smoking, CSF IL-1β, and multiple subdimensions of sleep, further elucidating the interaction between smoking and CSF IL-1β through moderation analysis.

## 2 Materials and methods

### 2.1 Participants

All participants in this study were recruited from a population of patients undergoing lower extremity surgery at several hospitals in Inner Mongolia due to ligament injuries or fractures below the knee, between September 2014 and January 2016. The study included 191 Chinese adult males aged 17–64 years, comprising 104 non-smokers and 87 active smokers. Demographic data, including age and BMI, were recorded. Exclusion criteria were as follows: (1) a family history of psychosis or neurological diseases; (2) systemic or CNS diseases as determined by the Mini International Neuropsychiatric Interview. Non-smokers were individuals who had never smoked and had no history of substance abuse or dependence. Active smokers were defined as those who consumed at least half a pack of cigarettes (i.e., 10 cigarettes) per day for more than 1 year, according to the Diagnostic and Statistical Manual of Mental Disorders, 4th Edition. Smokers consuming fewer than 10 cigarettes per day were excluded. None of the participants had a history of alcohol abuse, psychiatric disorders, or other substance abuse. This study was conducted in accordance with the Declaration of Helsinki (World Medical Association, 2008) and approved by the Institutional Review Board of Inner Mongolian Medical University (YKD2014031). All participants provided written informed consent, and no financial compensation was provided for their involvement in the study.

### 2.2 Assessments, biological sample collection, and laboratory tests

The Chinese version of the Pittsburgh Sleep Quality Index (PSQI) is a 9-item self-report measure assessing sleep over the previous month, which has demonstrated good reliability and validity among Chinese adults (Tian et al., 2024; Tsai et al., 2005). Items were rated on a 4-point Likert scale, where 0 represents “no difficulty" and 3 indicates “severe difficulty." The PSQI assesses the following subdimensions of sleep: subjective sleep quality, latency, duration, habitual efficiency, disturbances, use of sleep medication, and daytime dysfunction. The PSQI total score ranges from 0 to 21, with higher scores indicating poorer sleep. The PSQI was administered prior to the lumbar puncture surgery by researchers who have undergone unified training.

Lumbar puncture, a routinely preoperative procedure for anterior cruciate ligament reconstructive surgery in China, facilitated CSF sample collection, minimizing the influence of disease on the sample. Preoperative smoking cessation was not mandated for this surgery. Anterior cruciate ligament reconstruction typically lasted under 1 h, with a maximum interval of two days between hospitalization and surgery. A licensed anesthetist performed the lumbar puncture in the morning using 3 mL of 0.5 % ropivacaine for local anesthesia, yielding a 5 mL CSF sample via intrathecal collection. Each cerebrospinal fluid sample was then distributed into ten 0.5 mL tubes and immediately frozen at −80 °C for storage, without undergoing centrifugation. CSF IL-1β levels were measured by the human IL-1β ELISA kit (7.8–1000 pg/mL, Cloud-clone Corp., USA), according to the manufacturer's instructions. Laboratory personnel were blinded to the clinical data.

### 2.3 Statistical analysis

The Shapiro–Wilk test was used to assess normality, revealing non-normal distributions for age, BMI, PSQI scores, and CSF IL-1β levels. Consequently, the Mann–Whitney *U* test was used to compare demographic data, PSQI scores, and raw CSF IL-1β levels between active smokers and non-smokers. Spearman correlation analysis was conducted to examine correlations between CSF IL-1β levels and subdimensions of sleep. Linear regression analyses evaluated the main effects of smoking and IL-1β on sleep subdimensions, as well as the moderation effect of smoking on the relationship between IL-1β and sleep. Each regression model included age and BMI as control variables in the first step, smoking and IL-1β as main effects in the second step, and the smoke × IL-1β interaction term in the third step. Simple slope analyses were used to further examine significant moderation effects. Statistical analyses were conducted using R Programming Language version 4.2.0 and the R package, with significance set at p < 0.05 for all tests.

## 3 Results

### 3.1 Descriptive statistics

Descriptive statistics for variables in this study are presented in Table 1. Active smokers exhibited significantly higher values for age (34.39 ± 10.49 vs. 29.56 ± 9.49, p < 0.001), BMI (25.88 ± 3.59 vs. 24.91 ± 4.04, p < 0.05), and PSQI total scores (4.30 ± 2.52 vs. 2.85 ± 2.47, p < 0.001) compared to non-smokers. Moreover, active smokers had significantly higher scores for sleep quality, sleep latency, sleep efficiency, and sleep disturbance (all p < 0.05) compared to non-smokers. However, no significant differences were observed in sleep duration, sleep medication, daytime dysfunction scores, or CSF IL-1β levels between active smokers and non-smokers.

**Table 1:** Comparisons of age, BMI, PSQI total scores, subdimensions of sleep, and CSF IL-1β levels between non-smokers and active smokers.

Variable	Non-smokers	Active smokers	*P*
	Mean ± SD (n = 104)	Mean ± SD (n = 87)	
**Age (years)**	29.56 ± 9.49	34.39 ± 10.49	<0.001∗∗∗
**BMI (kg/m^2^)**	24.91 ± 4.04	25.88 ± 3.59	0.02∗
**PSQI Total Scores**	2.85 ± 2.47	4.30 ± 2.52	<0.001∗∗∗
**Subdimensions of Sleep**			
1.Sleep Quality	0.55 ± 0.63	0.74 ± 0.61	0.03∗
2.Sleep Latency	0.38 ± 0.64	0.66 ± 0.62	<0.001∗∗∗
3.Sleep Duration	0.68 ± 0.71	0.82 ± 0.85	0.39
4.Sleep Efficiency	0.09 ± 0.38	0.37 ± 0.86	0.02∗
5.Sleep Disturbance	0.43 ± 0.57	0.79 ± 0.50	<0.001∗∗∗
6.Sleep Medication	0.05 ± 0.25	0.02 ± 0.15	0.54
7.Daytime Dysfunction	0.65 ± 0.77	0.91 ± 0.93	0.07
**IL-1β (pg/mL)**	11.0 ± 4.70	10.53 ± 6.54	0.07

Note: BMI, body mass index; PSQI, Pittsburgh Sleep Quality Index; IL-1β, Interleukin-1β. Statistical comparisons were performed by using Mann-Whitney U tests between non-smokers and active smokers. ∗p < 0.05, ∗∗p < 0.01, ∗∗∗p < 0.001.

### 3.2 Correlation analysis between IL-1β and subdimensions of sleep

**Fig. 1:**
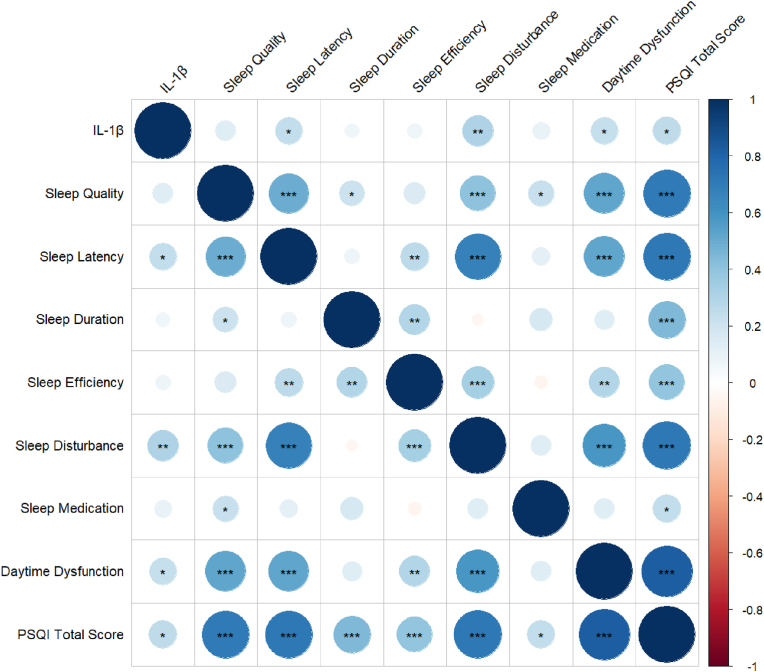
Correlation analysis between IL-1β and subdimensions of sleep among non-smokers. Note: IL-1β, Interleukin-1β; PSQI, Pittsburgh Sleep Quality Index. All data were reported using Spearman correlation analysis. ∗p < 0.05, ∗∗p < 0.01, ∗∗∗p < 0.001.

Table 2 and Fig. 1 illustrate the correlations between CSF IL-1β levels and subdimensions of sleep among non-smokers. Results revealed CSF IL-1β levels were positively correlated with sleep latency, sleep disturbance, daytime dysfunction, and PSQI total scores among non-smokers (results for subdimensions of sleep: latency: r = 0.24, p < 0.05; sleep disturbance: r = 0.31, p < 0.01; daytime dysfunction: r = 0.24, p < 0.05; PSQI total scores: r = 0.25, p < 0.05). Conversely, among active smokers, no significant correlation was found between CSF IL-1β levels and PSQI total scores (see Supplementary Table 1 and Supplementary Fig. 1). Correlation analysis between CSF IL-1β levels and subdimensions of sleep revealed lower CSF IL-1β levels in active smokers were significantly associated with poorer sleep efficiency (r = −0.26, p < 0.05), no other subdimensions of sleep reached statistical significance (see Supplementary Table 1 and Supplementary Fig. 1).

**Table 2:** Correlation analysis between IL-1β and subdimensions of sleep in non-smokers.

	IL-1β	Sleep Quality	Sleep Latency	Sleep Duration	Sleep Efficiency	Sleep Disturbance	Sleep Medication	Daytime Dysfunction	PSQI Total Scores
IL-1β	1								
Sleep Quality	0.14	1							
Sleep Latency	0.24 ∗	0.50 ∗∗∗	1						
Sleep Duration	0.07	0.21 ∗	0.08	1					
Sleep Efficiency	0.07	0.15	0.27 ∗∗	0.3 ∗∗	1				
Sleep Disturbance	0.31 ∗∗	0.4 ∗∗∗	0.67 ∗∗∗	−0.05	0.34 ∗∗∗	1			
Sleep Medication	0.10	0.22 ∗	0.11	0.17	−0.06	0.14	1		
Daytime Dysfunction	0.24 ∗	0.53 ∗∗∗	0.52 ∗∗∗	0.13	0.30 ∗∗	0.58 ∗∗∗	0.14	1	
PSQI Total Scores	0.25 ∗	0.71 ∗∗∗	0.71 ∗∗∗	0.44 ∗∗∗	0.40 ∗∗∗	0.72 ∗∗∗	0.25 ∗	0.82 ∗∗∗	1

Note: IL-1β, Interleukin-1β; PSQI, Pittsburgh Sleep Quality Index. All data were reported using Spearman correlation analysis. ∗p < 0.05, ∗∗p < 0.01, ∗∗∗p < 0.001.

### 3.3 Moderation analysis

**Fig. 2:**
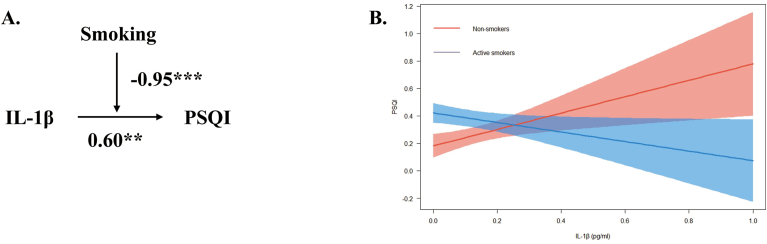
Moderation effect of smoking on the relationship between IL–1β and PSQI total score. Note: IL-1β, Interleukin-1β; PSQI, Pittsburgh Sleep Quality Index. (A) Conceptual model of moderation analysis regarding smoking as moderator. (B) Simple slope analysis for the moderation effect of smoking on the relationship between IL-1β and PSQI scores. The model was adjusted by age and BMI. ∗p < 0.05, ∗∗p < 0.01, ∗∗∗p < 0.001.

**Fig. 3:**
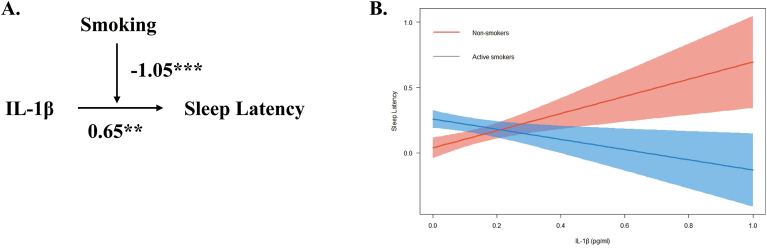
Moderation effect of smoking on the relationship between IL–1β and sleep latency score. Note: IL-1β, Interleukin-1β. (A) Conceptual model of moderation analysis regarding smoking as moderator. (B) Simple slope analysis for the moderation effect of smoking on the relationship between IL-1β and sleep latency. The model was adjusted by age and BMI. ∗p < 0.05, ∗∗p < 0.01, ∗∗∗p < 0.001.

**Fig. 4:**
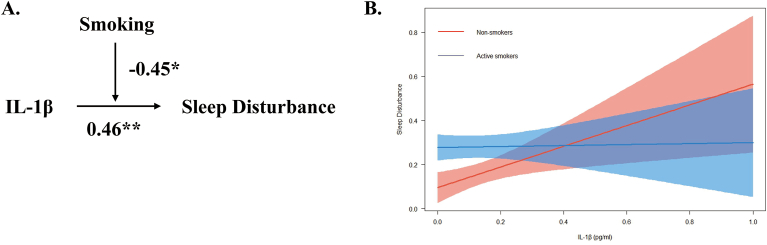
Moderation effect of smoking on the relationship between IL–1β and sleep disturbance score. Note: IL-1β, Interleukin-1β. (A) Conceptual model of moderation analysis regarding Smoking as moderator. (B) Simple slope analysis for the moderation effect of smoking on the relationship between IL-1β and sleep disturbance. The model was adjusted by age and BMI. ∗p < 0.05, ∗∗p < 0.01, ∗∗∗p < 0.001.

Moderation analyses were conducted based on the aforementioned correlations (see Table 2) and regression results below (see Table 3, Table 4, Table 5). These analyses aimed to investigate whether smoking moderates the effect of neuroinflammatory factor CSF IL-1β on PSQI sleep latency and sleep disturbance scores. Moderation effects were observed in PSQI scores, sleep latency, and sleep disturbance (refer to Fig. 2; Fig. 3; Fig. 4).

**Table 3:** Linear regression analysis for the moderation effect of smoking on the relationship between IL–1β and PSQI total score.

	Model 1(PSQI)		Model 2(PSQI)		Model 3(PSQI)	
	β	t	β	t	β	t
**Age**	0.21∗∗	2.74	0.15	1.92	0.18	2.37
**BMI**	0.19∗	2.05	0.15	1.67	0.16	1.80
**Smoking**	–	–	0.11	3.21	0.24∗∗∗	4.77
**IL-1β**	–	–	−0.01	−0.07	0.60∗∗	2.7
**Smoking∗IL-1β**	–	–	–	–	−0.95∗∗∗	−3.45
**R^2^**	0.05		0.09		0.14	
**F**	5.92∗∗		5.71∗∗∗		7.22∗∗∗	

Note: BMI, body mass index; PSQI, Pittsburgh Sleep Quality Index; IL-1β, Interleukin-1β. Model 1 was a linear regression model including age and BMI as the independent variables, with PSQI as the dependent variable. Model 2 was another linear regression model that included smoking and IL-1β as the independent variables, with PSQI as the dependent variable. Model 3 included smoking, IL-1β, smoking × IL–1β terms as independent variables and PSQI as the dependent variable. Both Model 2 and the Model 3 were adjusted for age and BMI. ∗p < 0.05, ∗∗p < 0.01, ∗∗∗p < 0.001.

**Table 4:** Linear regression analysis for the moderation effect of smoking on the relationship between IL-1β and sleep latency.

	Model 1(Sleep Latency		Model 2(Sleep Latency)		Model 3(Sleep Latency)	
	β	t	β	t	β	t
**Age**	0.16∗	2.31	0.12	1.69	0.16∗	2.23
**BMI**	0.06	0.75	0.04	0.45	0.05	0.56
**Smoking**	–	–	0.08∗	2.35	0.22∗∗∗	4.7
**IL-1β**	–	–	−0.01	−0.11	0.65∗∗	3.18
**Smoking∗IL-1β**	–	–	–	–	−1.05∗∗∗	−4.1
**R^2^**	0.02		0.04		0.11	
**F**	2.97		2.92∗		5.90∗∗∗	

Note: BMI, body mass index; IL-1β, Interleukin-1β. Model 1 was a linear regression model including age and BMI as the independent variables, with sleep latency as the dependent variable. Model 2 was another linear regression model that included smoking and IL-1β as independent variables, with sleep latency as the dependent variable. Model 3 included smoking, IL-1β, smoking × IL-1β terms as independent variables and sleep latency as the dependent variable. Both Model 2 and the Model 3 were adjusted for age and BMI. ∗p < 0.05, ∗∗p < 0.01, ∗∗∗p < 0.001.

**Table 5:** Linear regression analysis for the moderation effect of smoking on the relationship between IL–1β and sleep disturbance.

	Model 1(Sleep Disturbance)		Model 2(Sleep Disturbance)		Model 3(Sleep Disturbance)	
	β	t	β	t	β	t
**Age**	0.08	1.27	−0.01	−0.09	0.01∗	0.15
**BMI**	0.04	0.51	0.0001	0.001	0.003	0.05
**Smoking**	–	–	0.12∗∗∗	4.47	0.19∗∗∗	4.46
**IL-1β**	–	–	0.17	1.57	0.46∗∗	2.55
**Smoking∗IL-1β**	–	–	–	–	−0.45∗	−1.99
**R^2^**	−0.001		0.04		0.11	
**F**	0.94		2.92∗		5.54∗∗∗	

Note: BMI, body mass index; IL-1β, Interleukin-1β. Model 1 was a linear regression model that included age and BMI as independent variables, with sleep disturbance as the dependent variable. Model 2 was another linear regression model that included smoking and IL-1β as independent variables, with sleep disturbance as the dependent variable. Model 3 included smoking, IL-1β, smoking × IL-1β terms as independent variables and sleep disturbance as the dependent variable. Both Model 2 and Model 3 were adjusted for age and BMI. ∗p < 0.05, ∗∗p < 0.01, ∗∗∗p < 0.001.

Linear regression analysis indicated a positive relationship between CSF IL-1β and PSQI total scores (β = 0.60, p < 0.01). Smoking also had a significant effect on PSQI total scores (β = 0.24, p < 0.001). Furthermore, smoking negatively moderated the relationship between CSF IL-1β and PSQI scores (β = −0.95, p < 0.001; see Table 3).

Similar significant interactions were observed in several subdimensions of sleep. IL-1β was associated with increased sleep latency scores (β = 0.65, p < 0.01), while smoking also exhibited a positive association with sleep latency scores (β = 0.22, p < 0.001). Moderation analysis revealed that smoking significantly moderated the relationship between IL-1β and sleep latency scores. (β = −1.05, p < 0.001; see Table 4).

Furthermore, IL-1β was found to influence sleep disturbance scores (β = 0.46, p < 0.01), indicating a positive association with higher disturbance levels. There was a positive relationship between smoking and sleep disturbance scores. (β = 0.19, p < 0.001). Moderation analysis revealed smoking significantly moderated the relationship between IL-1β and sleep disturbance scores. (β = −0.45, p < 0.05; see Table 5).

Conceptual models illustrating the moderation effect for the aforementioned moderation effects were created respectively (see Fig. 2A; Fig. 3A; Fig. 4A). Simple slope analyses were conducted to further examine the significant moderation effects to investigate the impact of IL-1β on total PSQI scores (see Fig. 2B), sleep latency (see Fig. 3B), and sleep disturbance (see Fig. 4B) for non-smoker and active smoker groups.

## 4 Discussion

The study aimed to elucidate the relationship between CSF IL-1β, smoking, and their effects on sleep given both inflammatory factors and smoking have been associated with sleep disorders in recent studies (Jehan et al., 2018; Zagaria et al., 2022). Results indicated active smokers exhibited higher PSQI total scores compared to non-smokers, whilst no difference was observed in CSF IL-1β levels between the two groups. Notably, among active smokers, no significant correlation between CSF IL-1β levels and PSQI total scores was observed. Conversely, non-smokers exhibited a positive correlation between CSF IL-1β levels and PSQI total scores, suggesting that IL-1β plays an important role in the circadian regulation of the central nervous system. Further analysis revealed positive correlations between CSF IL-1β levels and sleep latency, sleep disturbance, and daytime dysfunction among non-smokers. In contrast, a negative correlation was found between CSF IL-1β levels and sleep efficiency scores amongst active smokers. Specifically, our study found that low CSF IL-1β levels in active smokers were significantly associated with poorer sleep efficiency. Moderation analysis revealed a significant interaction between CSF IL-1β levels and smoking, suggesting smoking disrupted the physiological relationship between CSF IL-1β and the circadian regulation of the central nervous system. Additionally, the moderating effect extended to sleep latency and sleep disturbance, indicating that active smokers with high CSF IL-1β levels experienced shorter sleep latency and milder sleep disturbance compared to non-smokers with comparable IL-1β levels.

The current investigation revealed exacerbated sleep quality, prolonged sleep latency, impaired sleep efficiency, and heightened sleep disturbance among active smokers compared to non-smokers. These observations aligned with previous findings regarding poor sleep in active smokers from a different study (Liu et al., 2020). The detrimental impact of cigarette smoking on sleep has been well-documented (Purani et al., 2019), consistent with our findings of higher PSQI total scores in active smokers. However, sleep disorder encompasses multiple dimensions, necessitating an exploration of differential associations between specific sleep dimensions and smoking to develop a comprehensive theoretical framework. Our study corroborates previous research indicating smoking's association with specific sleep problems such as poor sleep quality, increased sleep latency, and shorter sleep duration (Albqoor and Shaheen, 2021). This relationship may be attributed to smoking and nicotine disrupting sleep structure, potentially by increasing alpha power and the dose-dependent relationship between smoking intensity and the reduction of electroencephalogram delta power during non-REM sleep (Truong et al., 2021). Additionally, previous studies have also reported that smoking may affect the sleep-wake cycle (Salín-Pascual et al., 2003).

CSF IL-1β levels were associated with several subdimensions of sleep, including sleep latency, sleep disturbance, and daytime dysfunction, particularly among non-smokers. IL-1β, as a mediator of inflammatory responses, has been shown to be related to various brain functions, including sleep regulation (Ballesteros-Zebadua et al., 2014; Taishi et al., 2012). However, the association between IL-1β and sleep disorders, as well as the upregulation of CSF IL-1β, remains inconclusive. While some studies suggest that sleep disorders are associated with increased levels of pro-inflammatory molecules, including IL-1β (LaVoy et al., 2020), others report conflicting results. Our study further indicated the relationship between CSF IL-1β and sleep differed between smokers and non-smokers, suggesting that smoking may modulate the relationship between CSF IL-1β levels and sleep.

An important finding of this study was the moderating effect of smoking on the relationship between CSF IL-1β levels and sleep. From a physiological perspective, our results suggest that smoking disrupts the physiological relationship between CSF IL-1β and sleep. Based on previous findings, we speculate while acute nicotine exposure increases IL-1β levels; chronic nicotine exposure may lead to a decrease in IL-1β levels. This may be associated with an immunologic “tolerant" state caused by prolonged inflammatory exposure (Esquivel et al., 2014; Razani-Boroujerdi et al., 2011), or it could be due to a reduction in IL-1β release from astrocytes due to chronic nicotine exposure (Westerlund et al., 2013). Animal studies also suggested that nicotine inhibits microglial cell proliferation, leading to a significant decrease in IL-1β levels (Guan et al., 2015). The specific mechanism by which nicotine modulates the immune/inflammatory system through the central nervous system to affect sleep remains inconclusive, and further research and validation are needed. Our study provides insights regarding this subject matter, suggesting that smoking may influence sleep by interfering with the central neuroinflammatory factor, CSF IL-1β. Furthermore, our findings revealed that the interaction between CSF IL-1β and smoking primarily affects sleep latency and sleep disturbance, with no significant impact on other subdimensions of sleep.

## 5 Limitations

Our study has several limitations. First, the participants were patients with anterior cruciate ligament injuries, which may have confounded our results compared to those from healthy individuals. Second, we exclusively recruited male subjects due to the scarcity of female smokers in China, warranting further investigation into gender differences in future research. Third, we did not assess the participants’ lifestyle in detail. As such we are unable to statistically assess or provide further insights regarding the impact of such differences on the results. Fourth, although the majority of participants in this study reported minimal use of sleep medication (with only six participants indicating 1–2 uses of sleep medication per week), we cannot discount the possibility that participants may have accessed and used sleep-promoting supplements.

## 6 Conclusion

The current study observed that smoking disrupts multiple subdimensions of sleep and moderates the effect of neuroinflammatory factor CSF IL-1β on PSQI sleep latency and sleep disturbance scores.

## CRediT authorship contribution statement

**Yueling Hu:** Writing – original draft, Formal analysis. **Siyuan Li:** Writing – original draft. **Yingjie Chen:** Writing – original draft. **Xingguang Luo:** Writing – review & editing. **Yu-Hsin Chen:** Resources. **Yimin Kang:** Resources. **Weiming Hu:** Resources. **Li Chen:** Resources. **Siling Ye:** Resources. **Xinchen Zhou:** Resources. **Yanlong Liu:** Writing – review & editing. **Fan Wang:** Writing – review & editing. **Yuying Li:** Writing – review & editing, Writing – original draft.

## Ethics statements

The present study was approved by the Institutional Review Board of Inner Mongolian Medical University (Ethical approval number: YKD2014031), and was performed in accordance with the Declaration of Helsinki. The participants provided their written informed consent to participate in this study.

## Funding

This work was supported by the following grants: The Technology Support Project of Xinjiang (2017E0267), the 10th Inner Mongolia Autonomous Region "Prairie excellence" Project (FW), Natural Science Foundation of Xinjiang Province (2018D01C228), Xinjiang Outstanding Youth Science Grant (2017Q007).

## Declaration of competing interest

We declare that there are no conflicts of interest regarding this research manuscript. We have no financial, personal, or professional interests that could be construed as influencing the work presented in this paper.

## Data Availability

Data will be made available on request.
