# Supplementary material for: Smoking disrupts the relationship between cerebrospinal fluid IL-1β and multiple subdimensions of sleep
Source: Brain Behav Immun Health. 2025 Mar 26;45:100987. doi: 10.1016/j.bbih.2025.100987 (PMC11998107; doi:10.1016/j.bbih.2025.100987)
Supplement: Multimedia component 1 [file mmc1.docx]

**Supplementary Table 1**

Correlation analysis between IL-1β and subdimensions of sleep among active smokers.

|  | IL-1β | Sleep Quality | Sleep Latency | Sleep Duration | Sleep Efficiency | Sleep Disturbance | Sleep Medication | Daytime Dysfunction | PSQI Total Scores |  |
| --- | --- | --- | --- | --- | --- | --- | --- | --- | --- | --- |
|  |  |  |  |  |  |  |  |  |  |  |
| IL-1β | 1 |  |  |  |  |  |  |  |  |  |
| Sleep Quality | 0.01 | 1 |  |  |  |  |  |  |  |  |
| Sleep Latency | -0.17 | 0.08 | 1 |  |  |  |  |  |  |  |
| Sleep Duration | 0.01 | 0.16 | -0.01 | 1 |  |  |  |  |  |  |
| Sleep Efficiency | -0.26* | -0.01 | 0.22* | 0.49*** | 1 |  |  |  |  |  |
| Sleep Disturbance | -0.09 | 0.03 | 0.27* | -0.05 | 0.08 | 1 |  |  |  |  |
| Sleep Medication | -0.05 | 0.18 | -0.03 | 0.06 | 0.1 | 0.07 | 1 |  |  |  |
| Daytime Dysfunction | -0.05 | 0.25* | 0.08 | 0.12 | 0.25* | 0.34** | -0.06 | 1 |  |  |
| PSQI Total Scores | 0.14 | 0.71 *** | 0.71 *** | 0.44 *** | 0.40 *** | 0.72 *** | 0.25 * | 0.82 *** | 1 |  |

Note: IL-1β, Interleukin-1β; PSQI, Pittsburgh Sleep Quality Index. All data were reported using Spearman correlation analysis. *p<0.05, **p<0.01, ***p<0.001.


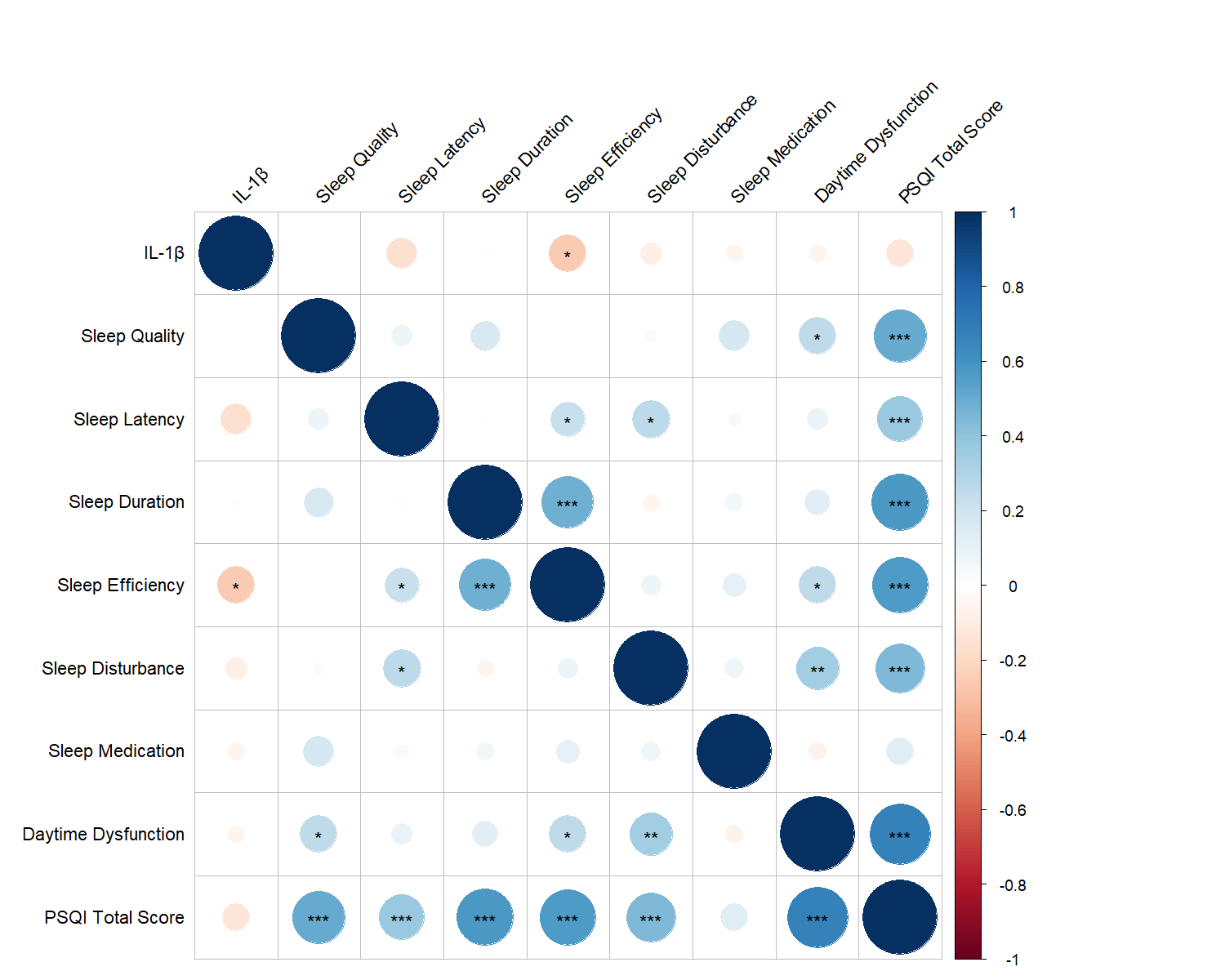


**Supplementary Fig. 1.** Correlation analysis between IL-1β and subdimensions of sleep among active smokers.

Note: IL-1β, Interleukin-1β; PSQI, Pittsburgh Sleep Quality Index. All data were reported using Spearman correlation analysis. *p<0.05, **p<0.01, ***p<0.001.
